# Sigmoid volvulus: definitive surgery is safe and should be considered in all instances

**DOI:** 10.1007/s11845-021-02713-0

**Published:** 2021-07-29

**Authors:** Niall P. Hardy, Philip D. McEntee, Paul H. McCormick, Brian J. Mehigan, John O. Larkin

**Affiliations:** Department of Colorectal Surgery, St James’s Hospital, Dublin 8, Ireland

**Keywords:** Endoscopic decompression, Preventative surgery, Recurrent volvulus, Sigmoid volvulus, Surgery

## Abstract

**Background:**

Acute sigmoid volvulus (ASV) represents a small but significant portion of cases of large bowel obstruction, especially in the elderly and co-morbid. Given the characteristics of the patient cohort most commonly affected, a non-operative/conservative approach is often undertaken but is associated with a high rate of recurrence.

**Objective:**

We sought to evaluate outcomes for those patients who underwent non-operative management, emergency surgery or staged, semi-elective surgery following decompression for ASV at our institution.

**Methods:**

Hospital in-patient enquiry (HIPE) data were used to identify all patients who presented with sigmoid volvulus between January 2005 and June 2020 inclusive. Patient notes were interrogated, including surgical and endoscopic procedures performed. Patient demographics and co-morbidities were recorded.

**Results:**

Thirty-nine patients were treated over a 15-year period with a mean age of 73 years at first presentation (range 36–93). Twenty-two patients (56%) had just a single admission for ASV with three deaths in this group.

Seventeen patients (44%) had more than one admission with volvulus due to recurrence after a decompression-only strategy on the index admission. Of these, three succumbed to complications of their subsequent episodes of volvulus.

Twenty-five patients underwent surgical intervention (fifteen on, or shortly following, their first admission and ten following at least two admissions for ASV). The overall mortality in the operative group was 2/25 (8%) with both deaths in those undergoing emergency surgeries. Five patients were treated successfully with endoscopic measures alone and had required no further interventions at the time of compiling data.

**Conclusion:**

There is a high recurrence rate following non-operative management of acute sigmoid volvulus and consequently, a cumulative increase in the attendant significant morbidity and mortality with subsequent episodes. Given the relatively low complication rate of definitive surgery, even in those patients perceived to be high risk, we contend that all patients should be considered for early surgery to prevent the likely recurrence of sigmoid volvulus.

## Introduction

Acute sigmoid volvulus occurs when a long or redundant segment of the sigmoid colon twists upon its own mesentery resulting in colonic obstruction and, if uncorrected, eventually bowel ischaemia [1, 2]. It is most predominant in Indian, African and Middle Eastern countries, although it is seen worldwide with a predilection for the elderly and infirm. It most commonly affects males with peak incidences between the 6th and 8th decades [3–5]. Diagnosis has traditionally been made on plain film imaging of the abdomen with the classic coffee bean appearance of the twisted sigmoid colon in the right upper quadrant; however, this has been mostly superseded by computed tomography in modern day evaluation of bowel obstruction as the diagnostic modality of choice [6, 7].

The American Society for Colon and Rectal Surgeons and American Society for Gastrointestinal Endoscopy both recommend initial endoscopic decompression of all haemodynamically stable patients, without evidence of perforation or ischaemia, followed by urgent definitive surgery (either restorative or non-restorative) as recurrence rates have been shown to be up to 90% with decompression alone [8–10]. Despite this, the often co-morbid status of the patient cohort involved, and sometimes reluctance from patient’s family members, may increase the temptation to defer definitive surgery. As the population continues to age, and increasingly complex and co-morbid patients are being considered for surgery, ASV is likely to become a more commonly encountered general surgery emergency. Given this, we sought to review our unit’s experience of the operative and non-operative management of this condition.

## Methods

Following insitutional ethical approval via the “Research and Innovation Office”, hospital in-patient enquiry (HIPE) data were used to identify all patients who presented to St James’s Hospital Dublin with sigmoid volvulus between January 2005 and June 2020. The diagnosis was established on radiologic, endoscopic or operative findings. Patient notes were examined, including surgical procedures and all endoscopic procedures performed. Patient demographics and co-morbidities were recorded. Elective surgery was defined as planned surgery following successful endoscopic decompression or surgery at a later, separate, admission. Mortality was defined as death within 30 days of hospitalisation.

## Results

Thirty-nine patients were treated for sigmoid volvulus over a 15.5-year period January 2005–June 2020 inclusive. Of these thirty-nine patients 25 were male and 14 female. The mean age at time of initial presentation with sigmoid volvulus was 73 years (range 36–93). Thirteen of the patients (33%) were residents of a nursing home or long-term care facility at the time of their initial presentation (Table 1).

**Table 1:** Patient demographics for all individuals admitted with ASV from 2005 to 2020

Patient demographics	
**Age** (years) *n* = 39	
Mean	72.9 (SD 14.19)
Range	36–93
Median	76
**Sex**	
Male *n* = 25	Female *n* = 14

**Fig. 1:**
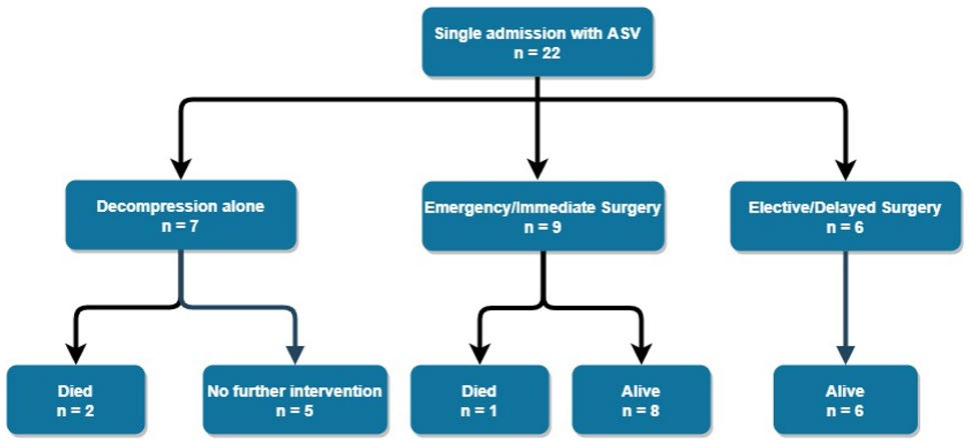
Management of patients with a single admission for acute sigmoid volvulus

Twenty-two (56%) patients during this time period had just a single admission with three deaths amongst this group (Fig. 1). One individual died eight days post-admission for ASV. A successful endoscopic decompression was performed on presentation; however, the patient developed worsening renal function progressing to multiorgan failure ultimately resulting in their demise at day eight. Similarly, a second patient aged 84 underwent successful endoscopic decompression but died the following day from respiratory failure and worsening acidosis. Neither patient was noted to have ischaemic bowel at endoscopy. The third death occurred in a 74-year-old man who presented with CT confirmed sigmoid volvulus and proceeded to immediate laparotomy. Intra-operatively, more extensive large bowel ischaemia was found to be present, and a total colectomy with end ileostomy was performed. The patient developed significant respiratory sepsis, on a background of severe COPD, post-operatively, and died on day 17 post-admission.

Fifteen patients underwent surgery (9 emergency and 6 elective) on, or shortly following, their first admission with one mortality as mentioned. Indications for emergent surgery included one or more of the following: failure of endoscopic decompression, evidence of mucosal ischaemia at endoscopy and radiological evidence of ischaemia (with or without haemodynamic instability). Elective surgery was considered in cases where successful endoscopic decompression, or improvement in clinical status, permitted patient optimization and involvement of colorectal subspecialty input.

Five patients with just a single admission each for ASV were treated successfully with endoscopic decompression alone and had had no further admissions to our institution at the time of compiling data.

**Fig. 2:**
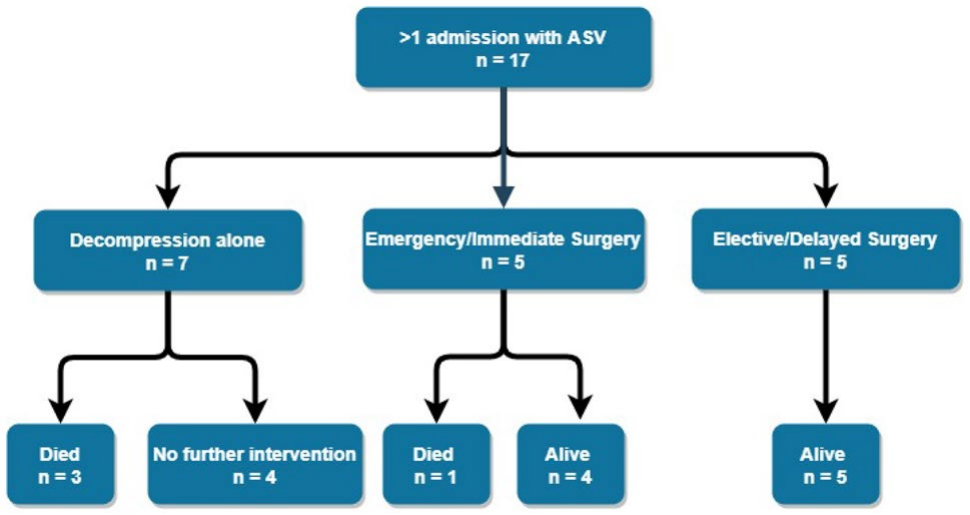
Management of patients with > 1 admission for acute sigmoid volvulus

Seventeen (44%) patients had two or more admissions for ASV (Fig. 2). Ten underwent surgery on their final admission (5 emergency and 5 elective) with one mortality in the emergency surgery group. Three patients with at least one previous admission with a conservatively managed volvulus ultimately died as a result of volvulus recurrence. These three patients had 3, 4 and 14 separate admissions, respectively, all for sigmoid volvulus.

**Fig. 3:**
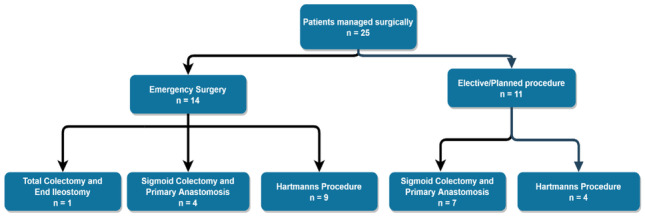
Surgical management of patients by operation type

The overall mortality in all patients undergoing surgery was 8% (2/25), and both deaths were in patients undergoing emergency surgery (Fig. 3).

The mean number of admissions for sigmoid volvulus for those managed non-operatively following the first presentation was 4.43 (range 2–14).

Seven patients with more than one admission had a higher ASA grading on their final admission when compared to their initial presentation, including the one patient who died after emergency surgery on a repeat admission for ASV (Table 2).

The average number of admissions for all patients within the study was 2.18.

**Table 2:** Patient number and ASA grade stratified by management approach

ASA score (at last presentation)	Patients treated with emegency surgery (number)	Patients treated with staged surgery (number)	Patients treated with decompression alone (number)
2	5	6	3
3	6	4	8
4	2	1	3
5	1	0	0

### Surgical management (Fig. 3)

Twenty-five of the thirty-nine patients underwent surgery, elective or emergency, with two deaths. Thirteen underwent a Hartmann’s procedure, and eleven had a sigmoid colectomy and primary anastomosis. Of those who received a primary anastomosis, seven were in the staged/semi-elective surgery cohort and four in the emergency surgery cohort. One patient in the emergency surgery group underwent a subtotal colectomy and end ileostomy but later died from respiratory sepsis. The only other mortality was in a patient who underwent an emergency Hartmann’s procedure on their second admission for recurrent volvulus. The initial post-operative period was uneventful and the patient was discharged on post-operative day 11. Unfortuately, the patient re-presented to the emergency department the following day having collapsed and died secondary to a myocardial infarction.

In those undergoing elective surgery, only one patient had a complication in the form of a sinus tract at the lower end of the laparotomy scar which required debridement and re-suturing.

Amongst the emergency surgery cohort, one patient (ASA 3) suffered wound dehiscence requiring resuturing, one patient had a protracted ileus which settled with supportive management, three patients developed a lower respiratory tract infection and one patient developed a self-limiting, per rectal bleed from a colorectal anastomosis managed with transfusion of red cells. There were no anastomotic leaks.

## Discussion

Sigmoid volvulus represents a small but significant portion of all cases of large bowel obstruction, especially in the elderly and co-morbid. Treatment of ASV with endoscopic decompression alone, with or without the insertion of a flatus tube, has been shown to have good short-term results but with a high recurrence rate and this is reflected in our unit’s experience [11, 12].

Although limited by its relatively small, retrospective and single-centre nature, our data support a more aggressive surgical approach when dealing with this patient cohort with good outcomes. Operative approach can be left to the operating surgeon’s discretion allowing for intra-operative findings, condition of the patient and experience/specialty of the surgeon involved. Our study findings, however, support the use of resection and primary anastomosis where deemed appropriate, especially in the setting of planned elective surgery. The safety of primary anastomosis has also been demonstrated elsewhere with similar results [12–14].

Johansson et al. reported a series of 168 patients with ASV and concluded that given 20% of their cohort did not experience a recurrence after their first episode that an ‘endoscopic alone’ strategy could be taken in cases of first presentation [15]. We note however a considerable mortality rate in those that represented with > 1 episode of ASV (4/17) compared with no mortalities in those that underwent delayed surgery following a successful endoscopic detorsion. A recent survey on contemporaneous practice amongst American Society of Colon and Rectal Surgeons members found that 81.2% of 197 respondents would consider a sigmoid colectomy on index admission for successful endoscopic detorsion [16]. Seventy-four percent of those surveyed were colorectal specialists, however, and given the nature of ASV as an emergency presentation, this finding may not accurately represent the approach of all sub-specialties on the general surgery call rota.

It should also be noted that, of the patients in our study who presented more than once with ASV, seven were found to have a higher ASA score at the time of their final presentation when compared to their index presentation, including the one mortality in the emergency surgery group. Given the well-established inverse relationship between ASA and outcome at emergency laparotomy, this finding further supports definitive management at the time of initial presentation [17, 18]. Nonetheless, when taking into account the broadly comparable ASA figures across the operative and non-operative groups (see Table 2), we argue that outcomes remain optimal for those treated with definitive surgery irrespective of ASA.

Whilst mortality is the ultimate adverse outcome from sigmoid volvulus, the significant impact on quality of life and morbidity that recurrent sigmoid volvulus has on a patient is worth considering. As is evident from the results of this study, patients treated non-operatively spent many hospital days undergoing repeat endoscopic interventions. Indeed, our figures depicting the number of patients treated “successfully” (deemed almost universally as patient survival) may even potentially overestimate success rates as some of these patients may have been admitted elsewhere for treatment unknown to the authors of this study.

An important mention should also be given to another similar yet distinctly separate cause of abdominal pain presenting in a similar fashion that mimics ASV: colonic pseudo-obstruction [19]. Whilst the underlying pathology in ASV is mechanical, pseudo-obstruction likely represents autonomic dysfunction in the absence of any physical obstruction, although the exact pathophysiology is debated [20]. These patients may also respond well initially to endoscopic decompression; however, given they do not have the same ischaemic risk that ASV patients do, surgical intervention is not as often required and, instead, should be substituted for more conservative measures such as laxatives, intensive nursing care in the form of frequent turning or mobilisation and a review of potential exacerbating medications.

A small number of patients will present *in extremis* at first presentation requiring emergency surgical intervention or may not even survive to reach specialist care. A significant proportion of patients, however, will have had previous admissions with the condition, and it is here that improvements in patient care can be made. These improvements can translate to reduced mortality rates but also to fewer admissions and the requirement for fewer invasive, temporising methods of management such as decompressive sigmoidoscopy.

## Conclusion

There is a high recurrence rate following non-operative management of acute sigmoid volvulus and consequently a cumulative increase in the attendant significant morbidity and mortality with subsequent episodes. Given the relatively low complication rate of definitive surgery, even in those patients perceived to be high risk, we contend that all patients should be considered for early surgery to prevent the likely recurrence of sigmoid volvulus.

## Data Availability

Data available on request.
